# Ryanodine receptors

**DOI:** 10.1186/2044-5040-1-18

**Published:** 2011-05-04

**Authors:** E Michelle Capes, Randall Loaiza, Héctor H Valdivia

**Affiliations:** 1 Department of Cellular and Regenerative Biology, University of Wisconsin Medical School. Madison, WI 53711, USA

## Abstract

Excitation-contraction coupling involves the faithful conversion of electrical stimuli to mechanical shortening in striated muscle cells, enabled by the ubiquitous second messenger, calcium. Crucial to this process are ryanodine receptors (RyRs), the sentinels of massive intracellular calcium stores contained within the sarcoplasmic reticulum. In response to sarcolemmal depolarization, RyRs release calcium into the cytosol, facilitating mobilization of the myofilaments and enabling cell contraction. In order for the cells to relax, calcium must be rapidly resequestered or extruded from the cytosol. The sustainability of this cycle is crucially dependent upon precise regulation of RyRs by numerous cytosolic metabolites and by proteins within the lumen of the sarcoplasmic reticulum and those directly associated with the receptors in a macromolecular complex. In addition to providing the majority of the calcium necessary for contraction of cardiac and skeletal muscle, RyRs act as molecular switchboards that integrate a multitude of cytosolic signals such as dynamic and steady calcium fluctuations, β-adrenergic stimulation (phosphorylation), nitrosylation and metabolic states, and transduce these signals to the channel pore to release appropriate amounts of calcium. Indeed, dysregulation of calcium release via RyRs is associated with life-threatening diseases in both skeletal and cardiac muscle. In this paper, we briefly review some of the most outstanding structural and functional attributes of RyRs and their mechanism of regulation. Further, we address pathogenic RyR dysfunction implicated in cardiovascular disease and skeletal myopathies.

## Introduction

In striated and smooth muscle cells, fluctuations in the intracellular levels of Ca^2+ ^ions greatly determine the magnitude and duration of contractile force. In cardiac and skeletal muscle, depolarization of the external membrane and its invaginations, the T-tubules, elicits swift and massive Ca^2+ ^release from the sarcoplasmic reticulum (SR), which in turn causes 'flooding' of the contractile myofilaments with Ca^2+ ^and induction of contraction. This exquisitely coordinated series of events, in which an electrical stimulus (depolarization) is converted into a mechanical contraction, is collectively termed 'excitation-contraction (EC) coupling', and it has as central players the voltage-dependent Ca^2+ ^channels/dihydropyridine receptors (DHPRs) as the sarcolemmal voltage sensors, and the Ca^2+ ^release channels/ryanodine receptors (RyRs) as the SR Ca^2+ ^release conduits. The structural and functional communication between the voltage sensor and the RyR dictate the magnitude of Ca^2+ ^release from the SR, and thus the force of contraction. In fact, genetic mutations in either of these two proteins, or alterations in the environment that promotes their functional coupling, are known to cause ventricular arrhythmias, hypercontractures and/or pathological remodeling of cellular structures. Excellent reviews on DHPRs have appeared recently [1,2]. In the current review, we focus on RyRs to discuss their most prominent structural and functional attributes, and to suggest mechanisms by which their dysfunction leads to disease.

### Structural features of RyRs

#### RyR isoforms

RyRs are not restricted to striated muscle. This class of intracellular Ca^2+ ^release channels is also found in the endoplasmic reticulum of neurons, exocrine cells, smooth-muscle cells, epithelial cells, lymphocytes, sea-urchin eggs, and many others [3]. In all of these cells, RyRs play a central role in the regulation of the intracellular free Ca^2+ ^concentration ([Ca^2+^]_i_), whose elevation triggers a cascade of events that culminates in, for example, neurotransmitter release, hormone secretion, lymphocyte activation and egg fertilization. To gain the functional flexibility necessary to respond to different triggering signals, at least three isoforms of RyRs are expressed in mammals, each one encoded by a distinct gene (*ryr1*, *ryr2 *and *ryr3*), residing on separate chromosomes. RyR1 is expressed predominantly in fast- and slow-twitch skeletal muscle and at lower densities in cerebellar Purkinje cells, gastric smooth muscle and B lymphocytes, among others. RyR2 was originally purified from cardiac muscle (it is the major isoform expressed there), but is also robustly expressed in neurons, and in visceral and arterial smooth muscle. RyR3 is the least understood of the RyR isoforms and seems to play its most important role during development, although in mature cells RyR3 is found in diaphragm, epithelial cells, brain, and smooth muscle [3,4]. There is approximately 65% amino acid sequence identity between mammalian RyR isoforms [5]. Most prominent in sequence variation are three regions of divergence, termed D1, D2 and D3, corresponding to amino acids 4254 to 4631, 1342 to 1403, and 1872 to 1923 in RyR1, respectively to amino acids 4210 to 4562, 1353 to 1397, and 1852 to 1890 in RyR2, respectively. Because RyR2 cannot rescue EC coupling in dyspedic myoblasts (immature muscle cells lacking RyR1) [6], it is likely that these regions of divergence are responsible for the incapacity of rescuing EC coupling with either isoform. Indeed, D2 has been shown to play a crucial role in establishing mechanical coupling between RyR1 and the skeletal DHPR (Ca_v_1.1) [7], but a complete picture of all the structural elements participating in this mode of EC coupling is still lacking. D3, by contrast, contains a low-affinity Ca^2+ ^binding site [8], but whether this site corresponds to a Ca^2+ ^inactivation site that operates *in vivo *has not been determined.

#### Molecular architecture of RyRs

With a molecular weight in excess of 2 MDa, RyRs are indisputably the largest known ion channels. Although structural study of RyRs has been challenging because of the channel's large size, some details of RyR structure have been obtained through cry-oelectron microscopy (cryo-EM) [9-13], comparative modeling [14] and recently, x-ray crystallography of small RyR segments [15-17]. In electron micrographs, purified RyRs are seen as quatrefoil or cloverleaf-shaped structures [18-20], or in volume-filled renderings as mushroom-shaped structures, with a large (27 × 27 × 12 nm) cytoplasmic assembly and a smaller transmembrane 'stalk' spanning approximately 6.5 nm from the base of the cytoplasmic region and extending into the SR lumen [21]. The quatrefoil structure results from the symmetrical arrangement of four identical subunits of approximately 5,000 amino acids each, thus a single tetrameric channel encompasses approximately 20,000 amino acids. As if this structural assemblage was not massive enough, an RyR *in situ *serves as a structural scaffold for other proteins and cofactors that add to its discrete bulkiness and distinctive shape. Indeed, RyRs and associated proteins where first observed as the 'foot' structures that bridge the gap of approximately 12 nm that separates T-tubule and SR membranes in cardiac and skeletal muscle [22].

#### RyR channel protein assembly

The cytoplasmic assembly of RyRs consists of 15 distinct domains per subunit, including clamp-shaped domains around the periphery of the channel, connected to 'handle' domains surrounding a central cavity [23,24]. Single-particle reconstruction studies indicate that moving between the closed and open states involves conformational changes in the cytoplasmic assembly and in the transmembrane domains, which undergo dilation and constriction around the pore, similar to the iris of a camera diaphragm [25]. Molecular sieving experiments, streaming potential experiments, and electrical distance measurements have suggested a pore approximately 3 Å wide, with a voltage drop of approximately 10.4 Å in length [26]. RyR mapping indicates that the pore of the channel consists of six to eight transmembrane segments [27]. Recent studies using single-particle three-dimensional reconstructions of cryo-EM images of RYR1 at refined resolutions (approximately 10 Å) have enabled a more detailed picture of the secondary structure of the channel pore to be pieced together. Samso *et al. *[13] suggested that each monomer has at least six transmembrane α-helices, with four inner helices forming a 'tepee' around the central cavity. Further, in the closed state of the channel, the conformation of the inner helices of RyR1 resembles that of the closed KcsA channel. However, Ludtke *et al. *[28] identified only five helix-like densities lining the pore of the RyR1 channel, which, in its closed state, more closely resembles the structure of open MthK channels rather than closed KcsA channels. The authors conceded that higher resolution was needed for further clarification.

This incongruity was addressed in a later paper [11], in which the authors compared the open and closed conformation of the channel to produce three independent 3D reconstructions, all of which upheld the previous report of six transmembrane helices. Those authors found that relocation of the ion pathway, in which the inner helices bend outward and the inner branches separate slightly, is directly related to an increase of 4 Å in diameter of the pore during channel gating. Further, by superimposing the 3D reconstructions of the open and closed channel, they were able to identify structural hinges that correspond to the previously identified binding sites of regulatory proteins, including calmodulin (CaM) and FK506 (tacrolimus)-binding protein (FKBP)12. Thus, they suggested that the hinges may be the mechanism by which the binding of a small effector is transduced to the pore region.

**Figure 1:**
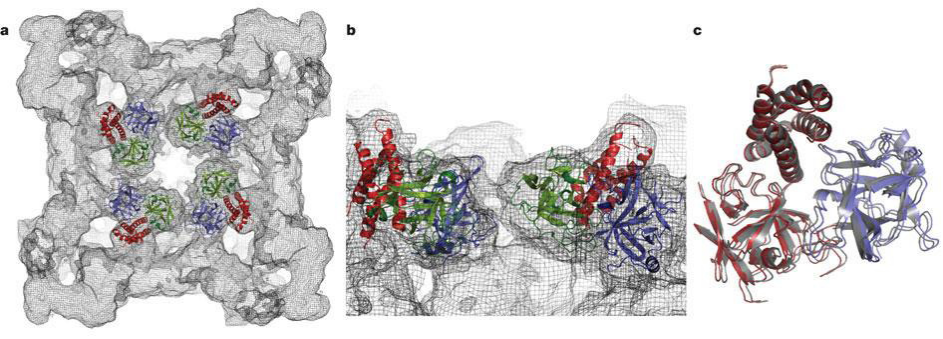
**Crystallized structure of rabbit ryanodine receptor (RyR)1 for amino acid residues 1-559**. This RyR1 segment folds into three distinct domains, forming a vestibule around the four-fold symmetry axis: domain A (blue; 1-205), domain B (green; 206-394) and domain C (red; 395-532). **(A) **Cytoplasmic view; **(B) **close-up lateral view from the four-fold symmetry axis, **(C) **Docking of domain A alone (blue) or domain BC alone (red) yields a near-perfect superposition on the docking position for ABC (grey). Adapted from Tung, *et al. *[17].

Two studies have recently solved high-resolution crystal structures (2.5 Å) of portions of the N-terminal region of the skeletal RyR (RyR1) associated with pathogenic mutations. The first study crystallized the N-terminal 210 residues, which were found to adopt a β-trefoil fold, similar to a suppressor domain of inositol trisphosphate receptors (IP_3_Rs) [15]. The disease mutations investigated in this study clustered in a region within a newly identified domain, highlighting its importance in channel regulation. The authors proposed that the β-trefoil fold, like the analogous structure in IP_3_Rs, may couple to the C-terminal channel domain to form a suppressor/coupling domain. The second study crystallized a larger construct, comprising N-terminal residues 1 to 559 [17]. This region folds into three distinct domains (arbitrarily termed A, B and C) per monomer (Figure 1), forming a vestibule around the pore of the channel. Fifty pathogenic RyR mutations have been mapped to this region, grouped on the basis of their proposed effect on channel function. Six of the mutations are buried deep within the domains, most probably causing protein misfolding rather than faulty interaction with channel modulators. Significantly, most mutations were mapped to inter-domain and inter-subunit interfaces, thus suggesting that RyR gating is allosterically coupled to the movement of the A, B and C domains, and agreeing well with the hypothesis proposed by Ikemoto *et al. *[29-31] that disruption of any of the inter-domain interfaces by disease-associated mutations may destabilize closed states, increasing the probability of the channel opening.

#### The RyR macromolecular complex

As mentioned above, RyRs are often complexed with several accessory proteins, forming an intricate multi-protein array [32,33]. The best known RyR-interacting proteins are CaM, which tonically inhibits RyR2 activity and produces biphasic effects on RyR1 [34,35]; FKBP12 and FKBP12.6, which stabilize RyR1 and RyR2 closures [36-38]; and the ternary complex triadin-junctin-calsequestrin, which 'senses' luminal Ca^2+ ^content and modulates RyR activity by acting either as a Ca^2+ ^reservoir or as a direct channel ligand [39-47]. More recently, RyR2 has been found to hold anchoring sites for protein kinase (PK)A, protein phosphatase (PP)1, the cAMP-specific phosphodiesterase (PDE)4D3 and Ca^2+^/calmodulin-dependent protein kinase (CaMK)II [37,48], emphasizing the importance of RyR2 regulation by phosphorylation [32]. In cardiac cells, sorcin exerts protein-protein interactions with the RyR and inhibits Ca^2+ ^release in a Ca^2+^-dependent manner [49,50].

The binding sites of several regulatory proteins have been mapped near the 'clamp' region of the channels, encompassing subdomains 5 to 10, an apparent hot-spot for allosteric modulation. Phosphorylation sites for CaMKII and PKA, including Ser2808 and Ser2030, occur in different subdomains within this region [51,52]. Muscular A kinase anchoring protein (mAKAP) is believed to bind to RyRs via leucine zippers at residues 3003 to 3039, tethering PKA close to these phosphorylation sites [32]. Another scaffolding protein, Homer, purportedly interacts with RyRs via the clamp domain, where it brings Ca^2+ ^signaling proteins in close proximity to their respective binding sites, and may facilitate crosstalk between RyRs and proteins in the surface membrane, such as DHPRs and β-adrenergic receptors [53-55].

CaM, both in its Ca^2+^-bound (Ca^2+^-CaM) and unbound forms (apoCaM), reportedly binds in a cleft between the 'clamp' and 'handle' regions [56]. This cleft occurs between domains 3 and 7 on a portion of the cytoplasmic assembly of the channel facing the SR, about 10 nm from the pore [57]. From their cryo-EM studies, Samso and Wagenknecht [57,58] proposed a model in which CaM transitions between a distinct, but overlapping apoCaM site as calcium concentration ([Ca^2+^]_i_) fluctuates. According to this model, as apoCaM binds Ca^2+ ^ions, it loses affinity for the apoCaM site and translocates to the nearby Ca^2+^-CaM site. The CaM binding sites identified by these studies was corroborated by a Förster resonance energy transfer (FRET)-based approach, which identified a CaM target helix that spanned residues 3614 to 3643. However, [Ca^2+^]-dependent translocation of CaM between adjacent or overlapping sites could not be detected by FRET, indicating that any such movements must be subtle. The authors pointed out that such structural rearrangements could also be explained by changes in the interaction of CaM with the RyR target sequence or by rotation of CaM around its major axis [59].

Collins originally reported that a 12-kDa immunophilin (FKBP12) copurified with RyR1 [60]. Jayaraman *et al. *later reported that FKBP12.0 binds to RyR1 with a stoichiometry of one molecule per tetrameric channel [61]. Experimental techniques similar to those described above have shown that the binding site of FKBP12 also occurs in the clamp region of the channels; however, there is some controversy about the exact amino acid region(s) responsible for this interaction. For RyR1 and RyR3, a valyl-prolyl motif (amino acid residues 2461 and 2462 in RyR1) seems to berequired for binding of FKBP12/FKBP12.6 [62,63]. For RyR2, a variant of this motif (isoleucyl-prolyl sequence 2427 and 2428, respectively) has been implicated in binding FKBP12.6 [37]. However, Masumiya *et al. *reported that mutation of Ile-2427 or Pro-2428 does not alter FKBP12.6 binding [64], and further, that the RyR2 polypeptide fragment encompassing residues 1 to 1855 is sufficient for binding of FKBP12.6 [65]. Zhang *et al. *also found that other regions of RyR2 contribute to FKBP12.6 binding [66]. As with CaM, Wagenknecht and colleagues [57] used 3D reconstructions of cryo-EM images of purified RyRs complexed with FKBP12 to investigate the site of the interaction, which was identified on the opposite side of domain 3 from CaM, about 12 nm from the pore. A later study compared 3D reconstructions of native and FKBP12-stripped RyR1, identifying two surfaces of interaction between FKBP12 and RyR1: a portion of the β-sheet of FKBP12 interacts with domain 9 of RyR1 and the intersection of domains 3, 5 and 9, while loop 87 to 90 interacts with domain 3 of RyR1 [12].

### Functional regulation of ryanodine receptors

In cardiac muscle, a small influx of external Ca^2+ ^(*I*_Ca_) through DHPRs (Ca_v_2.1) binds to and opens RyR2, releasing a larger amount of Ca^2+ ^from the SR. This process, known as Ca^2+^-induced Ca^2+ ^release (CICR [67,68]), occurs in diads, which are specialized regions in which a segment of a T-tubule seems to be continuous with a semi-spherical extension of the SR [22]. CICR amplifies the incoming Ca^2+ ^signal approximately 10 to 20 times, and is therefore the major component of the intracellular Ca^2+ ^transient that induces contraction. In skeletal muscle, *I*_Ca _is not required for contraction [69]. Instead, mechanical coupling between DHPRs (Ca_v_1.1) and RyR1 (direct or facilitated by intermediary proteins) triggers Ca^2+ ^release from the SR immediately after sarcolemmal depolarization [70,71]. At the triads, where a segment of one T-tubule seems to be continuous with two SR evaginations, not all RyRs are closely apposed to DHPRs; nearly half of RyRs remain DHPR-free and are therefore presumed to be activated by Ca^2+ ^release from neighboring RyRs, effectively amplifying the Ca^2+ ^signal [72]. Thus, common subcellular structures participate in EC coupling, but different processes link membrane depolarization to Ca^2+ ^release in cardiac and skeletal muscle. In this section, we review some of the most prominent regulators of RyR1 and RyR2 and their effect in Ca^2+ ^release in skeletal and cardiac muscle.

#### Ca^2+ ^regulation

All three RyR isoforms harbor both activating and inactivating Ca^2+ ^binding sites. Regardless of some differences (noted below), the Ca^2+ ^affinity for those sites is roughly similar for all RyRs. Thus, Ca^2+ ^plays a crucial role in the modulation of RyR activity, even in skeletal muscle [72].

**Figure 2:**
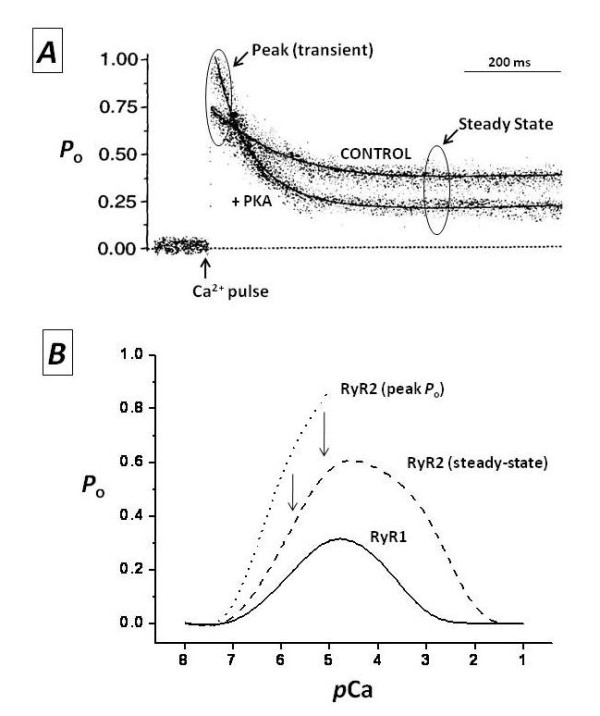
**Modulation of ryanodine receptors (RyRs) by Ca^2+^**. (**A**) Ensemble currents of single RyR2 show the effect of fast Ca^2+ ^pulses on the *P*_o _of a non-phosphorylated channel ('control') or the same channel after phosphorylation with the catalytic subunit of protein kinase A ('+PKA'). Laser photolysis of 'caged Ca^2+^' (o-nitrophenyl EGTA, tetrapotassium salt; NP-EGTA) increased [Ca^2+^] from 0.1 to 10 μmol/l at the point labeled as 'Ca^2+ ^pulse' (modified from [51]. **(B) **Ca^2+^-*P*_o _curves of RyR1 and RyR2. Channel activity was measured at the indicated stationary concentrations of Ca^2+ ^('RyR1' and 'RyR2 steady-state') and right after a fast Ca^2+ ^pulse, as in (A) ('RyR2 (peak *P*_o_)') (unpublished results) (see text for details).

The RyR is a Ca^2+^-gated channel, hence, functional assays that measure its activity ([^3^H]ryanodine binding, reconstitution in lipid bilayers, Ca^2+ ^sparks) require a critical [Ca^2+^] in the medium to maintain the channel in its open state. However, despite the fact that RyRs *in situ *normally encounter fast and transient changes in [Ca^2+^], most of the functional characterizations of single RyR have been obtained under the continuous presence of a critical [Ca^2+^], that is, under steady-state conditions. How similar is this characterization to that obtained under a transient application of [Ca^2+^]? Fabiato, in his classic studies delineating CICR [67,68], found that the amount of Ca^2+ ^release was strongly dependent on the velocity of the Ca^2+ ^pulse ((*d*[Ca^2+^]/*d*t) applied to skinned cardiac fibers. In light of these data, it is surprising that few studies have addressed the importance of dynamic fluctuations of Ca^2+ ^on RyR kinetics. Three such studies have revealed that a fast Ca^2+ ^stimulus elicits only a transient increase in the *P*_o _of RyRs; however, if Ca^2+ ^is applied rapidly but sustained for long periods, RyR *P*_o _will 'relax' to a new steady-state that follows a bell-shaped curve as a function of the magnitude of Ca^2+ ^stimulus. Thus, *P*_o_-[Ca^2+^] curves of greater magnitude emerge from titration of RyR activity with fast, calibrated Ca^2+ ^pulses than those obtained under similar but steady applications of Ca^2+ ^(Figure 2) [73-75]. The implication of these findings is that there are components of RyR activity that remain undetected in most of the assays presently used, and these aspects of RyR regulation may explain seemingly discrepant results, as in the case of RyR phosphorylation (see below).

Under steady-state conditions, Ca^2+ ^concentrations in the range of approximately 100 nmol/l to approximately 10 μm/l activate all three RyR isoforms [76-78]. The activation curve is sigmoidal, but the exact Hill number (cooperativity) and the plateau (maximal activation) depend greatly on the presence of other cytosolic ligands. Higher concentrations of Ca^2+ ^in the range of 100 μmol/l to approximately 10 mmol/l also inactivate the three RyR isoforms, although there is considerable RyR heterogeneity in this process [79,80]. In general, Ca^2+^-dependent inactivation is most prominent in RyR1 and less important in RyR2 and RyR3 [26]. In fact, complete Ca^2+^-dependent inactivation in isolated RyR2 channels occurs at such high [Ca^2+^] (>10 mmol/l) that its physiological relevance is questionable [58,76]. The agonist effect of ATP and the inhibitory action of Mg^2+ ^greatly influence the kinetics of calcium-dependent activation and inactivation [74], but the concentration of these two ligands is not expected to change dramatically over a short period or on a beat-to-beat basis, thus they may be considered rather as tonic modulators of RyRs whose influence becomes relevant under periods of intense muscle activity or in precarious metabolic states. Given the greater sensitivity of RyR1 to Ca^2+^-dependent inactivation, it is not surprising that Mg^2+ ^also exerts a more powerful inhibition on the RyR1 isoform [74,81], with Mg^2+ ^apparently binding to both Ca^2+ ^activation and inactivation sites, attenuating the former and intensifying the latter.

Recently, regulation of RyRs by luminal (intra-SR) Ca^2+ ^has been proposed as a critical control mechanism of Ca^2+ ^release, especially in cardiac cells. The crucial observation is that, beyond a certain threshold, small changes in SR Ca^2+ ^load result in far greater increases in Ca^2+ ^release, with the relationship described as an inverse hyperbole with high degree of cooperativity [82,83]. Direct Ca^2+ ^binding to sites available only from inside the SR [84], activation of cytosolic sites by the Ca^2+ ^ions being permeated by the channel ('feed-through' mechanism [85]), and most notably, regulation by the junctin-triadin-calsequestrin ternary complex (for example, [39-47]) remain as the leading candidate mechanisms effecting luminal Ca^2+ ^regulation of RyRs, although it has been difficult to dissect out the specific contribution of each of these phenomena.

#### RyR phosphorylation

RyRs are structural scaffolds for important kinases and phosphatases [32], and in the heart, RyR2 is one of the first proteins to undergo phosphorylation during β-adrenergic stimulation [86-88]. However, the functional effect of RyR phosphorylation is unclear and remains the subject of intense debate. All potential functional outcomes have been attributed to RyR phosphorylation. On one hand, Li *et al. *[89] found that PKA phosphorylation of RyR2 has little functional relevance for diastolic Ca^2+ ^release if SR Ca^2+ ^levels remain constant. On the other hand, other groups have suggested that PKA phosphorylation of RyR2 is so essential to intracellular Ca^2+ ^homeostasis that derangement of this process may be the basis for heart failure (HF) [37,90] and catecholaminergic polymorphic ventricular tachycardia (CPVT) episodes [91]. Between these two extremes, other results, mainly from *in vitro *experiments, imply that PKA phosphorylation increases [86,92], decreases [74,93] or has no effect [94] on RyR2 activity.

Several factors preclude an easy interpretation of phosphorylation results. First, RyRs contain multiple phosphorylation sites that, depending on their phospho-state, may attenuate or synergize the effect of the other sites, or may require prior phosphorylation to activate the whole protein. To date, three phosphorylation sites have been recognized: Ser2809 (mouse RyR2 nomenclature) was first identified by Witcher *et al. *[86] as a CaMKII site, and later, Wehrens *et al. *suggested it as the only PKA site [95], even though RyR2 from mice with genetic ablation of this site may still be phosphorylated by PKA [88]. Ser2815 and Ser2030 seem to be exclusively phosphorylated by CaMKII [96] and PKA [97], respectively. Second, as mentioned above, RyR activity is strongly dependent on the speed of Ca^2+ ^application, which in turn may greatly influence the overall effect of phosphorylation. For example, PKA phosphorylation of isolated RyR2 increases a transient component of activity (peak activation) but accelerates the rate of adaptation to a lower steady-state level of activity [74,88]. In cellular settings, this effect would translate into greater and faster rates of Ca^2+ ^release in response to a given amount of Ca^2+ ^entry. Elegant experiments in which SR Ca^2+ ^load and *I*_Ca _were kept constant showed that β-adrenergic stimulation of ventricular myocytes accelerates the rate of Ca^2+ ^release and has little effect on the magnitude of the [Ca^2+^]_i _transient [98]. Similarly, experiments in rat and mouse skeletal muscle fibers showed little or no effect of β-adrenergic stimulation on the macroscopic Ca^2+ ^transients [99]. A recent paper suggested that differences in the redox state of RyR could account for some the discrepancies seen [100]. Although appealing, this hypothesis still needs to be corroborated.

#### CaM

CaM regulates RyR activity both directly as a ligand, and indirectly as a cofactor for CaMKII. CaM binding to RyR1 increases channel activity at low [Ca^2+^], and decreases activity at high [Ca^2+^] [35]. Nevertheless, at the cellular level, this fine modulation might be negligible compared with the effect of other RyR regulators such as DHPR and calcium [101]. In skeletal muscle cells, CaM is proposed to protect RyR1 from oxidative stress and also work as an oxidative stress sensor (see below). RyR1 nitrosylation decreases CaM binding and increases channel activity [102]. The physiological importance of this mechanism is not clear, but it has been related to pathological processes [56]. In single RyR2 channels, only inhibitory effects by CaM have been shown [26]. CaM binding to both RyR1 and RyR2 can also be regulated by S100A1, a calcium-activated protein that competes with CaM for the RyR binding site [103-105]. Although the physiological beat-to-beat significance of this interaction is not yet clear, its chronic deregulation potentially plays a role in HF [106,107]. A recent study suggested that PKA phosphorylation decreases CaM binding, which would subsequently increase channel activity [108].

#### Oxidative stress

Oxidative modifications of thiol residues in free cysteines, such as S-nitrosylation, S-glutathionylation and disulfide oxidation, can modulate RyR1 and RyR2 [109-111]. The functional response of RyR may vary depending on the cysteine residue being modified and the type of oxidative species that targets it [110,112]. In addition, oxidative modifications can also affect the binding of accessory proteins. For instance, most data suggests that single RyR1 exposure to nitric oxide (NO) increases channel activity [102]. In skeletal muscle fibers, this effect is more evident in the presence of CaM, suggesting that s-nitrosylation of some residues produces CaM detachment and therefore reversal of the inhibitory effect of CaM over RyR [56]. Whether NO directly regulates RyR2 seems likely, but it is not completely clear. The NO donor

S-nitroso-N-acetyl penicillamine (SNAP), which targets several EC-coupling proteins, increases inotropy of cardiac myocytes at low concentrations, but decreases it at high concentrations [113,114]. It has been proposed that reduced glutathione (GSH) could quickly react with and scavenge NO in cardiac cells [111]. Under these circumstances, nitroso-GS or other small nitrosylated molecules would be responsible for RyR oxidation [111]. It is also possible that the close proximity of nitric oxide synthase (NOS)-3, xanthine oxidase and RyR2 in cardiac caveolae creates a microenvironment capable of directly nitrosylating RyR2 [114], although in physiological conditions the main targets of NOS seem to be other EC-coupling proteins [115].

### RyR dysfunctions

In this section, we describe the role of RyR dysfunction in the generation of genetic and acquired diseases afflicting skeletal and cardiac muscle.

#### Malignant hyperthermia

Mutations in *RYR1*, the gene encoding the skeletal isoform of RyRs, are associated with malignant hyperthermia (MH), a pharmacogenetic disease triggered by inhalational anesthetics or depolarizing muscle relaxants [116,117]. SR Ca^2+ ^'leak' or excessive Ca^2+ ^mobilization from the SR due to hyperactivity of RyR1 (especially at rest) seems to be the underlying mechanism for this syndrome [118], although increased resting [Ca^2+^] in human muscle has yet to be categorically demonstrated [119]. MH episodes are rare (approximately 1 in 10,000 surgeries [120]), but in their severest clinical presentation, susceptible individuals anesthetized with halothane or other volatile anesthetics suddenly develop muscle rigidity, hyperkalemia, arrhythmias, respiratory and metabolic acidosis, and an alarming increase in body temperature. Fulminant death inexorably follows unless body temperature is lowered and the excessive SR Ca^2+ ^leak is prevented by dantrolene sodium, which presumably blocks RyR1, but not RyR2 [121]. More than 100 different point mutations in human *RYR1 *have been identified as potential causes of this syndrome; however, mutations in other genes, particularly in the α_1 _subunit of the skeletal DHPR, have also been reported to generate MH episodes [122]. This syndrome therefore emphasizes the central role that RyRs and DHPRs play in EC coupling. Interestingly, the great majority of *RYR1 *mutations seem to be clustered in three 'hot spots', namely, near the N-terminal (Cys^35-^Arg^614^), the central (Asp^2129^-Arg^2458^) and near the C-terminal (Ile^3916^-Ala^4942^) domains of the channel, a clustering that is repeated in an analogous RyR2-associated syndrome [123] (see below).

Porcine stress syndrome (PSS), which also results from point mutations of *RYR1 *(most often R615C) [124], is a similar MH syndrome, but with a wider array of triggering events (e.g. exercise, parturition, overcrowding). Susceptible pigs have been invaluable models to elucidate the molecular basis of MH in humans. Caffeine, which sensitizes RyR1 to Ca^2+^, elicits contractures of muscle strips of MH pigs at a lower dose than that required in control muscle. Caffeine hypersensitivity is also seen in human MH [125], and constitutes the basis for a clinical test of susceptibility. The bell-shaped Ca^2+^-dependence of [^3^H]ryanodine binding of MH-susceptible pigs is wider than that of normal pigs, suggesting greater sensitivity of RyR1 to Ca^2+ ^activation and increased resistance to Ca^2+ ^inactivation [77]. At the single channel level, RyR1s from MH-susceptible pigs exhibit similar conductance to 'normal' RyR1s, but they display longer mean open time and require higher [Ca^2+^] for inactivation. All these findings point to greater Ca^2+ ^mobilization from RyR1-controlled pathways during resting and contracting periods, with the resultant overflowing of cytosolic compartments with Ca^2+^, and the ignition of multiple signaling mechanisms. Heat seems to stem from the rampant hydrolysis of ATP by the SR Ca^2+ ^pump and other active Ca^2+ ^transporters trying to reestablish Ca^2+ ^homeostasis. However, the pathway to MH-associated heat production may be much more complex, as shown by the finding that MH mutations cause not only Ca^2+ ^dysregulation, but also an increased release of cytokines (for example, interleukin 6). In sufficiently large quantities, these cytokines can be pyrogenic [126].

#### Central core disease

Central core disease (CCD) is another autosomal-dominant myopathy resulting from mutations in *RYR1 *[127,128]. Although some patients with MH may show clinical myopathies before an MH episode, histopathological lesions characterized by pale staining of the central area of muscle fibers are necessary to diagnose CCD. Non-progressive muscle weakness, hypotonia and motor deficiencies are present in most, but not all, patients with CCD [129]. Type 1 skeletal muscle fibers are preferentially affected, with central areas lacking mitochondria and their oxidative enzymes, thus staining poorly with basophilic dyes [130]. The cause for the disappearance of central mitochondria is unknown. A potential explanation is that the *RYR1 *mutation, as in MH, causes excessive Ca^2+ ^leak. In the periphery of the fiber, Ca^2+^-extrusion mechanisms would be sufficient to remove the excess Ca^2+^, but the central areas would remain vulnerable to local Ca^2+ ^gradients. Central mitochondria would, therefore, 'swallow' greater amounts of Ca^2+ ^than they could handle, leading to swelling and death [131].

An alternative model for core formation has been proposed, based on studies using a knock-in mouse heterozygously expressing a leaky-channel mutation in the RyR1 N-terminal region [118,132-135]. According to this model, Ca^2+ ^leak in the SR triggers the release of reactive oxygen and nitrogen species (ROS/RNS), causing RYR1 S-nitrosylation and glutathionylation, modifications that further enhance SR Ca^2+ ^leak and RyR heat sensitivity. The authors correlated the resulting cascade of deterioration with the formation of cores that progress through a series of histopathologically distinct stages as the mice age. They proposed that oxidative damage, Ca^2+^-dependent proteolysis, and extreme stretching of the myofilaments due to prolonged contracture in the presence of unsequestered Ca^2+ ^may all contribute to the progression of the disease. Importantly, these mice are prone to fulminant MH-like responses to heat challenge and halothane exposure [118,135].

An entirely different mechanism for the muscle weakness associated with CCD was revealed in recent studies using a mouse knock-in model expressing a heterozygous mutation in the RyR1 ion pore [132]. Muscle fibers from these mice exhibited reduced maximal twitch contraction, slowed rate of force development [136] and reduced global Ca^2+ ^transients [137]. Adult mice displayed weakened upper body and grip strength [138]. These results were attributed to reduced and slowed RYR1 Ca^2+ ^release, without change in either SR Ca^2+ ^content or RyR1 sensitivity to activation by voltage or caffeine [138]; this pathway to CCD-associated muscle weakness is known as EC uncoupling [132]. Unlike the leaky-channel knock-ins, these mice were not prone to developing MH-like symptoms.

Surprisingly, the same mutation produced dramatically different phenotypes in different strains of mice, experimental approaches, and expression systems [136-138]. Similarly, identical *RYR1 *mutations cause MH in some human patients and CCD in others. The mechanism(s) that leads to the different phenotypes remains a mystery. Very interesting, however, is the observation that patients with MH who lack clinical myopathies are more likely to have mutations at the N-terminus of *RYR1*, whereas those with clinical myopathies are likely to have mutations at the C-terminus [128]. Although these observations seem to suggest that mutation mapping could provide an attractively simple predictor of phenotype, this notion probably oversimplifies the complexity of the problem. Transition from MH to CCD is probably a function of the severity of Ca^2+ ^leakage, as the studies discussed above have shown that both C-terminal and N-terminal mutations are capable of producing this type of RyR1 dysfunction.

#### CPVT

CPVT is an autosomal-dominant inherited cardiac disease characterized by exercise- or stress-induced tachyarrhythmia episodes in the absence of apparent structural heart disease or prolonged QT interval [139,140]. The disease is rare but very malignant, often presenting for the first time in childhood and adolescence as syncopal events and/or sudden cardiac arrest. Multiple electrocardiographic irregularities (polymorphic) are simultaneously present in patients with this syndrome. More than 70 different mutations in *RYR2*, the gene encoding the cardiac isoform of RyRs, have been associated with CPVT [141], which is characterized by: a) more than two types of ventricular tachycardia morphologies, b) absence of underlying organic heart disease and c) absence of primary electrical disease (long QT, Brugada syndrome) [142]. CPVT usually occurs during intense exercise or acute emotional stress, and may lead to sudden cardiac death. As in MH, excessive Ca^2+ ^release from the SR, especially during diastole, seems to be the underlying mechanism that gives rise to the VT [143,144]. Enhanced diastolic Ca^2+ ^leak may overload the Na^+^/Ca^2+ ^exchanger, which generates an inward current as it extrudes the released Ca^2+^. The inward current, in turn, gradually depolarizes the cell to threshold, favoring delayed after depolarizations (DADs) [145]. During depolarization, lack of Ca^2+^-dependent inactivation of *I*_Ca _due to previous depletion of the SR leads to higher Ca^2+ ^entry and reloading of the SR, which triggers another DAD in the next beat. Successive repetition of this altered Ca^2+ ^cycle could result in paroxysmal tachycardia and arrhythmias even if only a few foci of ventricular cells are involved.

Although this hypothetical scheme logically relates RyR2 dysfunction with VT, it is unclear exactly what mechanism induces a group of apparently normal RyR2s to behave aberrantly and to generate sudden tachycardia. Because infusion of catecholamines also triggers CPVT, it is likely that activation of the β-adrenergic system plays an important role. In this regard, it has been suggested that phosphorylation of RyR2 by PKA, the kinase linking β_1_-adrenergic receptor activation to cellular effects, dissociates FKBP12.6 [37,90,91,146]), an accessory protein that presumably stabilizes RyR2 in the closed state. Phosphorylation of a mutant RyR2 would therefore remove a stabilizer from a channel on the verge of dysfunction, and would cause the pathogenic events described above. However, several studies have not found any evidence of FKBP12.6 dissociation in CPVT mutation-carrying RyR2 [147,148]. Thus, structural alterations of the RyR2 channel complex seem to be more important than dysregulation by accessory factors in the pathogenesis of CPVT. Consistent with this notion is the fact that CPVT-associated mutations of *RYR2 *occur in domains corresponding exactly to mutation-containing domains that give rise to MH [123,141]. Although some of these mutations are close to the apparent FKBP12.x-binding domain, the great majority are not. Recent studies point to a predominant role of Purkinje cells in the genesis of ventricular arrhythmias [149-151]. Whatever the triggering mechanism, SR Ca^2+ ^load and release seem to be crucial, because mutations in *CSQ2*, the gene encoding cardiac calsequestrin, an intraluminal Ca^2+ ^buffering protein that regulates RyR2 activity, also generate CPVT [152]. Accordingly, a prominent hypothesis, championed mainly by Chen and collaborators, proposes that cardiac myocytes have a threshold SR Ca^2+ ^load for spontaneous Ca^2+ ^release, and that CPVT-associated mutations decrease the sensitivity to luminal Ca^2+ ^(that is, decrease the threshold) [143,153]. Thus, during β-adrenergic stimulation, when SR Ca^2+ ^load increases as a result of enhanced Ca^2+ ^entry and uptake, CPVT mutant channels reach their threshold and generate spontaneous Ca^2+^-release waves, creating a substrate favorable for Ca^2+^-dependent arrhythmias. Alternatively, CPVT-related mutations could increase the SR load, making it easier to reach the threshold upon adrenergic stimulation [154].

#### HF

HF is a multifactorial syndrome characterized by contractile dysfunction and pathological myocardial remodeling. There is growing consensus that Ca^2+ ^mishandling in HF leads to altered gene transcription, resulting in maladaptive structural transformations (cardiac hypertrophy and then wall thinning) that eventually hamper basic cardiac function [155]. As with the genetic RyR dysfunctions discussed above, an enhanced diastolic Ca^2+ ^leak seems to be central in the pathogenesis of some forms of HF [156]. According to a prominent hypothesis spearheaded by Marx *et al. *[37], increased catecholamine levels in patients with HF activate cAMP-dependent PKA by binding to β_1_-adrenergic receptors. PKA, in turn, phosphorylates RyR2-Ser2808 and dissociates FKBP12.6, increasing the probability of channel opening by inducing the appearance of long-lasting subconducting states. In this scheme, RyR2 hyperphosphorylation (up to 800% of control [37]) causes the enhanced diastolic Ca^2+ ^leak. Unfortunately, this mechanism remains controversial. Although a few studies support some aspects of this mechanism, most groups have not been able to reproduce the central tenets of this hypothesis. Although Jiang *et al. *reported that PKA and the drug JTV519 both decreased FKBP12.6 binding to RyR2 [157], several other groups found that PKA phosphorylation of RyR2-S2808 neither dissociated FKBP12.6 nor substantially modified channel gating [88,89,94,97,158,159]. Ai *et al. *[160] reported that RyR2-S2808 phosphorylation was increased by approximately 50% in a rabbit model of HF; however, a direct test of PKA phosphorylation of RyR2 in canine and human HF yielded no difference compared with control, and a phospho-antibody against RyR2-S2808 detected equal phosphorylation levels in control and HF samples [158,161]. More recently, a mouse with genetic ablation of the RyR2-S2808 site (S2808A), supposedly stabilized in the closed state by close association with FKBP12.6, displayed normal β-adrenergic response and similar progression towards heart failure compared with wild-type mice [88,159]. Finally, FKBP12.0 dissociation of RyR1 is consistently reported to destabilize channel closure, but at least two groups failed to detect effects of FKBP12.6 dissociation of RyR2 [38,162]. Clearly, more studies are needed to resolve these conflicting results and clarify the potential role of FKBP12.6, SR Ca^2+ ^leak, and PKA phosphorylation of RyR2-S2808 in HF.

## Summary

In addition to providing the majority of the Ca^2+ ^necessary for contraction of cardiac and skeletal muscle, RyRs act as molecular switchboards that integrate a multitude of cytosolic signals such as dynamic and steady Ca^2+ ^fluctuations, β-adrenergic stimulation (phosphorylation), nitrosylation and metabolic states, and transduce these signals to the channel pore to release appropriate amounts of Ca^2+^. Furthermore, because Ca^2+ ^release is crucially modulated by luminal factors such as SR Ca^2+ ^content and protein-protein interactions, RyRs play an additional role as integrative switch-valves that offset cytosolic-luminal Ca^2+ ^imbalances. Thus, there is an ample margin to interfere with the activity of RyRs. In both experimental and natural conditions, such interference results in overt contractile dysfunction and gross morphological changes. MH, CCD and CPVT are among the most studied clinical presentations of RyR dysfunction, and the elucidation of the precise molecular mechanisms affected by this dysfunction is advancing with great strides.

## Competing interests

The authors declare that they have no competing interests.

## Authors' contributions

EM, RL and HV contributed equally to the writing of this review. All authors read and approved the manuscript.
